# Basal pharyngeal pumping elevated in *C. elegans* *mod-5* mutants

**DOI:** 10.17912/W2H59W

**Published:** 2017-02-08

**Authors:** Terra Hiebert, Adela Chicas-Cruz, Kathyrn McCormick

**Affiliations:** 1 NemaMetrix, Inc,. 44 W 7th Ave., Eugene, OR 97401 USA.

**Figure 1.**
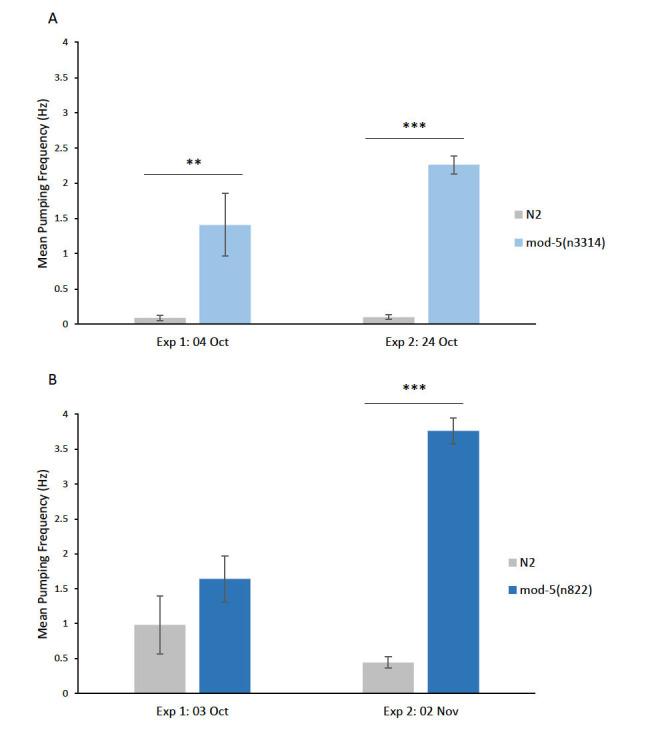


## Description

In *C. elegans*, the reuptake of serotonin (5-HT) is facilitated by *mod-5*, which encodes a 5-HT transporter that is orthologous to a human 5-HT transporter (SLC6A4). *mod-5* has been shown to effect both feeding and locomotion in *C. elegans* (Ranganathan et al., 2001; Jafarau et al. 2011). We obtained and analyzed EPG data using a microfluidic device (NemaMetrix) for *mod-5* null mutant strains, *mod-5*(*n3314*) (A, Exp 1 n=15; Exp 2 n=32) and *mod-5*(*n822*), (B, Exp 1 n=17; Exp 2 n=27) and N2 control worms (A, n=16 and 31; B, n=18 and 27 for Exp 1 and 2, respectively) in M9 saline buffer (2-minute recording duration). Mutations to the *C. elegans* serotonin reuptake transporter, *mod-5**,* lead to an accumulation of serotonin at the synaptic cleft, which results in a significant increase in baseline pharyngeal pumping frequency in three out of four experiments (A, N2=0.10 ± 0.04 and 0.09 ± 0.03 Hz ; *mod-5**(**n3314**)*=1.41 ± 0.44 and 2.26 ± 0.13 Hz; B, N2=0.98± 0.42 and 0.44 ± 0.08; *mod-5**(**n822**)*=1.64 ± 0.33 and 3.76 ± 0.19 Hz; **p<0.005, ***p<0.0001, 2-tailed students t-test).

## Reagents

Strains: MT8944: *mod-5**(**n822**)* I; MT9772: *mod-5**(**n3314**)* I Control Strain: N2
